# Successful transcatheter arterial embolization for uretero-inferior epigastric arterial fistula: A rare complication of cutaneous ureterostomy

**DOI:** 10.1016/j.eucr.2021.101726

**Published:** 2021-05-19

**Authors:** Rakuhei Nakama, Yasunori Arai, Yohei Takei, Tatsushi Kobayashi

**Affiliations:** Department of Diagnostic Radiology, National Cancer Center Hospital East. 6-5-1 kashiwanoha, Kashiwa, Chiba, 277-8577, Japan

**Keywords:** Uretero-arterial fistula, Transcatheter arterial embolization, Cutaneous ureterostomy, Inferior epigastric artery

## Abstract

A 70-year-old man presented with pulsatile bleeding upon changing his catheter for cutaneous ureterostomy. He was suspected to have a uretero-arterial fistula. Computed tomography showed an abnormally dilated right inferior epigastric artery, the suspected bleeding source. Angiography revealed a tortuous dilated branch from the inferior epigastric artery. Transcatheter arterial embolization was performed with a gelatin sponge and coil. He was discharged 15 days after the procedure. Uretero-arterial fistula is a rare but fatal complication among patients with long-term indwelling ureter catheters. An abnormal inferior epigastric artery surrounding the cutaneous ureterostomy should be considered a rare cause of uretero-arterial fistula.

## Highlights

• UAF is a rare but fatal complication in patients with indwelling ureter catheters.
• Delays in the diagnosis of UAF have caused poor outcomes.
• The aorta, and iliac and inferior epigastric arteries may cause UAF.
• The source of bleeding should be immediately identified using CT or angiography.
• Physicians should be aware of the rare causes of UAF.

## Introduction

Uretero-arterial fistula (UAF) is a rare but fatal complication in patients with long-term indwelling ureteral catheters. The fatality rate of UAF was reportedly around 20%, and massive bleeding was responsible for most fatalities.^1^

UAF is challenging to diagnose, and a delayed diagnosis can lead to poor outcomes.^2^ While pulsatile bleeding is suggestive of UAF, subsequent imaging cannot detect fistulas.^3^ Contrast-enhanced computed tomography (CT) may not be sensitive enough to identify bleeding signs, including pseudoaneurysm and extravasation.^4^

The aorta and iliac arteries near the ureter can develop fistulas.^3^ Therefore, these arteries are carefully evaluated on CT when UAF is suspected. However, UAF can also be caused by other arteries.

We present a case of UAF caused by the inferior epigastric artery as a rare complication of an indwelling ureteral catheter.

## Case presentation

**Fig. 1:**
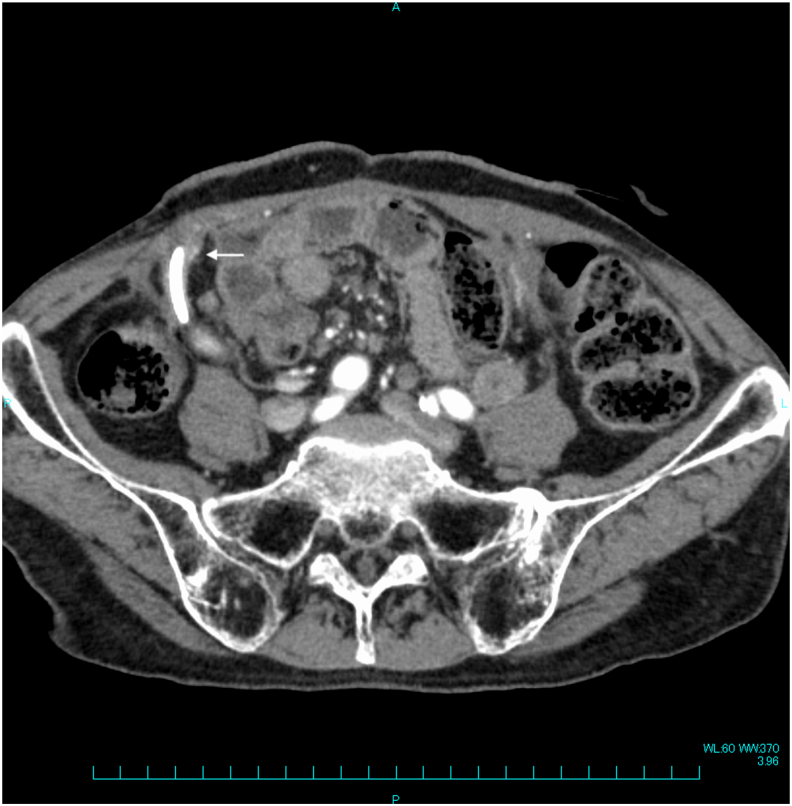
Contrast-enhanced computed tomography revealed an unnatural dilated right inferior epigastric artery located near his urethral catheter (arrow).

A 70-year-old man underwent total cystectomy and bilateral cutaneous ureterostomy for bladder cancer one year ago. He visited our hospital once a month to have his catheter changed for a cutaneous ureterostomy. He noticed intermittent hematuria for several days. When the urologists tried to change the catheter, pulsatile bleeding from the right cutaneous ureterostomy was observed. However, his hemodynamic status was stable, and laboratory tests showed no anemia or coagulopathy. His hemoglobin level was 9.3 g/dL, while the platelet count was 62.3 × 10^4^/μL. The prothrombin time-international normalized ratio was 1.06, while the activated partial thromboplastin time was 29.5 s. An uretero-arterial fistula was suspected. A contrast-enhanced CT results did not show an apparent communication between the aorta or iliac artery and the ureter. However, CT revealed an abnormally dilated right inferior epigastric artery surrounding the cutaneous ureterostomy without extravasation (Fig. 1). Angiography was performed to establish the diagnosis, and subsequent transcatheter arterial embolization (TAE) was done as needed.

**Fig. 2:**
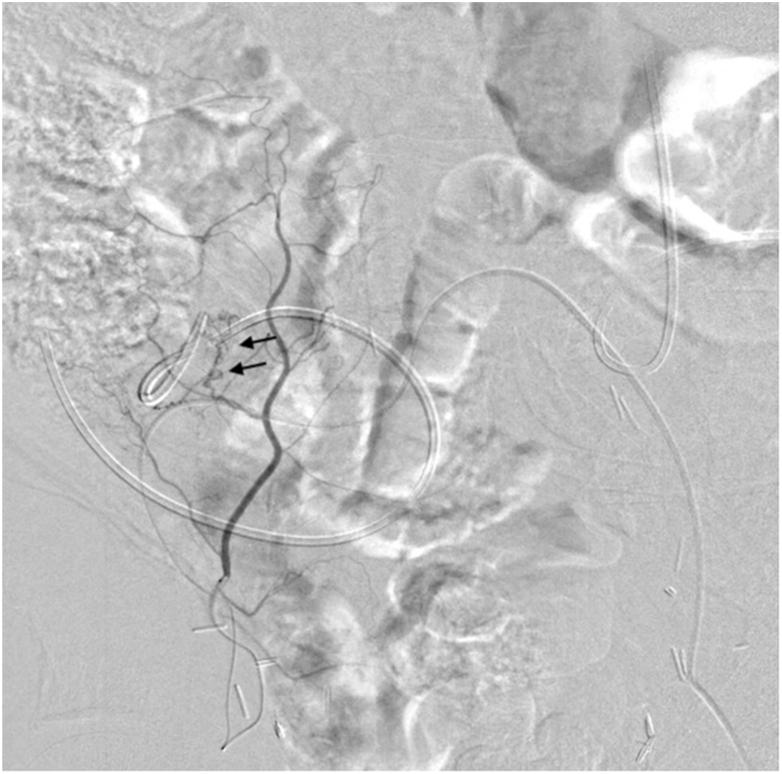
Angiography revealed a tortuous dilated branch from the right inferior epigastric artery (arrow).

The access route was secured with an 18-gauge needle and 4Fr catheter. A 25-cm sheath was created using the left femoral artery. The right external iliac artery was accessed using a 4Fr cobra-type catheter, and the right inferior epigastric artery branching near it was selected. We accessed the right inferior epigastric artery using a micro-guidewire (CHIKAI V, Asahi Intecc, Aichi, Japan) and a 1.5 Fr micro-catheter (Veroute ultra, Asahi Intecc, Aichi, Japan). Subsequent angiography revealed a tortuous dilated branch from the inferior epigastric artery, which was suspected to be the bleeding source (Fig. 2) as it was located near the ureteral catheter. We decided to embolize the artery from the bifurcation.

**Fig. 3:**
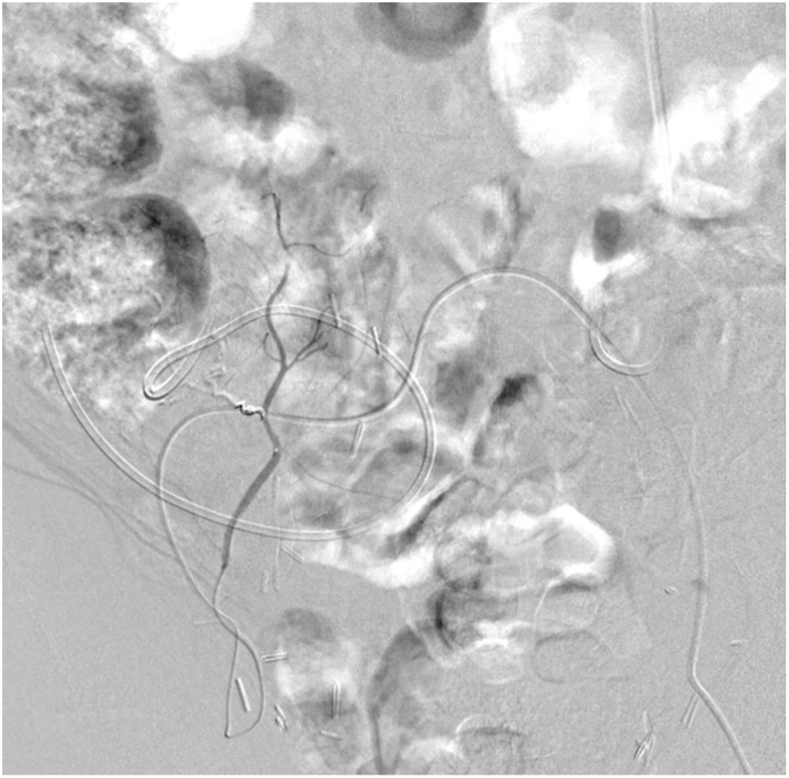
Final angiography via the right internal iliac artery showed the disappearance of the abnormal right inferior epigastric artery after embolization.

First, gelatin sponge particles (Serescue, Astellas Pharma, Tokyo, Japan) were injected until the blood flow decreased. Next, a micro coil (Hilal, Cook Medical Japan G. K., Tokyo, Japan) was placed at the bifurcation point. After embolization, angiography via the right internal iliac artery confirmed the disappearance of the tortuous dilated branch (Fig. 3).

No complications or recurrences of the bleeding from the cutaneous ureterostomy were not observed after TAE. The patient was discharged 15 days after the procedure.

## Discussion

We reported a case of uretero-inferior epigastric artery fistula, a rare complication of cutaneous ureterostomy, and an indwelling catheter. This patient was managed successfully because the CT findings were suggestive of UAF, and subsequent angiography and TAE achieved hemostasis before the patient developed hemorrhagic shock.

A case of uretero-inferior epigastric artery fistula has been previously reported,^5^ where CT revealed a dilated inferior epigastric artery near the cutaneous ureterostomy, which was suspected to be the cause of UAF, similar to the present case. Physical stress due to long-term catheter indwelling and exchange likely caused chronic inflammation in the cutaneous ureterostomy, resulting in vascular injury. When suspecting UAF in patients with cutaneous ureterostomy, the inferior epigastric artery should be carefully evaluated for abnormalities on CT.

In addition, angiography can be an excellent diagnostic tool for UAF.^4^ In our case, angiography revealed an abnormal tortuous inferior epigastric artery. Subsequent TAE safely and effectively controlled the bleeding. In particular, using a gelatin sponge to perform preventive localized embolization of the artery proximal to the ureteral catheter resulted in no active bleeding and is permissible.

## Conclusion

This case indicated that apart from the aorta and iliac artery, the inferior epigastric artery can also cause UAF. To avoid diagnostic delays, the source of bleeding should be immediately identified using CT or angiography. Interventional radiologists, urologists, and emergency physicians should be aware of the rare causes of UAF.

## Declaration of competing interest

None.
